# Arousal-Mediated Sleep Disturbance Persists During Cocaine Abstinence in Male Mice

**DOI:** 10.3389/fnins.2022.868049

**Published:** 2022-06-23

**Authors:** Theresa E. Bjorness, Robert W. Greene

**Affiliations:** ^1^Research Service, Veterans Affairs (VA) North Texas Health Care System, Dallas, TX, United States; ^2^Department of Psychiatry, Peter O’Donnell Jr. Brain Institute, University of Texas Southwestern, Dallas, TX, United States; ^3^Department of Neuroscience, Peter O’Donnell Jr. Brain Institute, University of Texas Southwestern Medical Center, Dallas, TX, United States; ^4^International Institute for Integrative Sleep Medicine, University of Tsukuba, Tsukuba, Japan

**Keywords:** sleep, cocaine, slow wave activity, arousal, mouse

## Abstract

Acute cocaine disturbs sleep on a dose-dependent basis; however, the consequences of chronic cocaine remain unclear. While the arousal promotion following cocaine has been well-established, effects of cocaine on sleep after termination of chronic cocaine exposure appear variable in human subjects with few studies in non-human subjects. Here, a within-subjects design (outcomes normalized to baseline, undisturbed behavior) and between-subjects design (repeated experimenter-administered cocaine vs. experimenter-administered saline) was used to investigate sleep homeostasis and sleep/waking under repeated cocaine/saline exposure and prolonged forced abstinence conditions in mice. Overall, during the forced abstinence period increases in arousal, as determined by sleep latency and gamma energy, persisted for 2 weeks. However, the sleep response to externally enforced sleep deprivation was unchanged suggesting that sleep disruptions during the forced abstinence period were driven by enhancement of arousal in the absence of changes in sleep homeostatic responses.

## Introduction

Cocaine, as a psychostimulant, acutely increases behavioral arousal and induces cortical desynchrony (Zubrycki et al., 1990; Kiyatkin and Smirnov, 2010). These behavioral and electrophysiological signatures of arousal are predominately caused by blockade of the dopamine transporter (DAT; Wisor et al., 2001); however, blockade of other monoamines may also contribute. As a strong arousal-promoting agent, cocaine induces sleep disturbance when taken during or near the habitual sleep period. In rodents, acute (as defined by a single exposure) cocaine dose-dependently increases sleep latency (Hill et al., 1977; Bjorness and Greene, 2018; Dokkedal-Silva et al., 2020) followed by a rebound sleep response with sleep/waking behavior normalizing within ∼24 h (Gruner et al., 2009; Bjorness and Greene, 2018). Conversely, chronic cocaine in which exposure occurs repeatedly across days, weeks, months, or years has inconsistent effects on sleep. For example, in rodents receiving experimenter-injected cocaine daily for 10 days, the sleep disturbance-sleep rebound pattern persisted (Dugovic et al., 1992), while in non-human primates self-administering cocaine, measures such as sleep efficiency showed tolerance over time (Cortes et al., 2016) suggesting time or experience-dependent effects independent of the direct pharmacological action. Inconsistent sleep-related outcomes are also observed in human subjects during both active use and withdrawal/abstinence periods (for review, Bjorness and Greene, 2021). This inconsistency in sleep-related outcomes following chronic cocaine may be due to a variety of methodological issues, such as timing relative to the sleep period, route of administration, polydrug exposure or history, and the population used as the control to name a few. Furthermore, individual differences may also lead to inconsistent sleep behavior-related effects since baseline sleep behavior (Webb and Campbell, 1983; Tang et al., 2007), response to sleep deprivation (SD; Franken et al., 2001; Van Dongen et al., 2012), and locomotor and reinforcing response to cocaine vary across individuals (Gulley et al., 2003; Belin et al., 2011; Perry et al., 2013).

Sleep behavior is controlled by two interacting processes; the circadian rhythm, which is modulated by cocaine and other drug exposure (Jansen et al., 2012; Stowie et al., 2015) and the sleep homeostat in which sleep pressure progressively increases across protracted waking (Borbely, 1982; Tobler and Borbely, 1986). Spectral power in the slow wave activity band (SWA; 0.5–4.5 Hz) during slow wave sleep (SWS, named for the appearance of this characteristic spectral activity) is the most sensitive index of the sleep homeostat often showing a “rebound” above typical baseline levels of SWS SWA following SD (Borbely et al., 1984). Thus far, the influence of chronic cocaine on sleep homeostasis as measured by SWS SWA has not been tested.

The influence of cocaine on sleep behavior may be functionally relevant for reward/addiction outcomes. Sleep disruption increases risk of relapse to cocaine use during abstinence in humans (Angarita et al., 2014), while SD enhances the rewarding properties of psychostimulants across species (Roehrs et al., 1999 [methylphenidate in humans]; Berro et al., 2018 [amphetamine in rats]; Bjorness and Greene, 2020 [cocaine in mice]). SD also increases motivation to self-administer cocaine in high-taking rats (Puhl et al., 2013) and expedites the incubation of cocaine craving (Chen et al., 2015).

The major advantage of the within-subjects design is that it mitigates possible innate differences in sleep/waking behavior prior to drug exposure between individual mice, while the major advantage of the between-subjects design is that it controls for the various non-pharmacological manipulations of the experiment (repeated handling, IP injections) which could influence sleep/waking behavior independently of direct psychostimulation.

Here, we used within-subjects (mice compared to their own baseline, undisturbed sleep behavior) and between subjects (saline exposure) in order to: (1) test the hypothesis that chronic cocaine alters the homeostatic response to prolonged waking and (2) determine the time course of possible sleep disturbance/sleep normalization across repeated cocaine exposure through several weeks of forced abstinence. We hypothesized that repeated cocaine would impair sleep homeostasis based on the shift from homeostasis to allostasis under chronic SD (Kim et al., 2012; Clasadonte et al., 2014). We did not formulate specific hypotheses to the particular degree of sleep disturbance and its pattern across time, though sleep disturbance during the cocaine exposure period and recovery during the 35 days forced abstinence period was expected based on reports of sleep/waking behavior at various time points in cocaine-dependent human users and cocaine-experienced animals (for review, Bjorness and Greene, 2021).

## Materials and Methods

**FIGURE 1:**
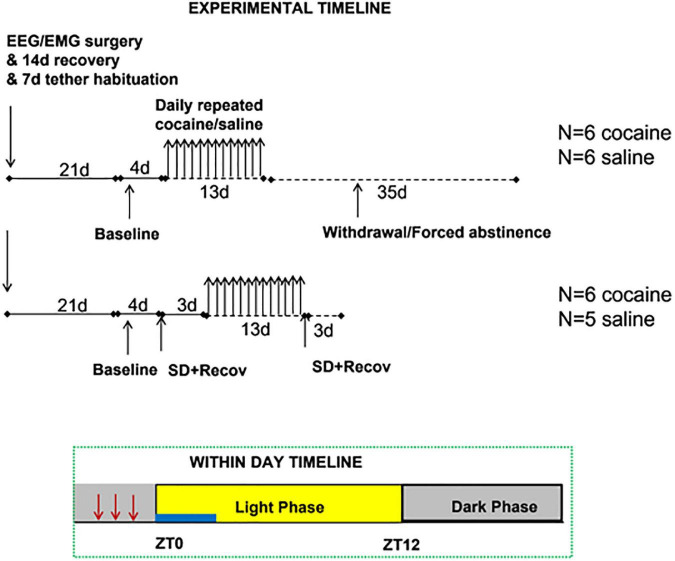
Experimental timeline: [Top] consisted of surgical implantation and recovery followed by baseline, cocaine/saline exposure (injection period), and 35 days forced abstinence (post-injection period), [Bottom] consisted of the same surgical implantation and recovery, followed by baseline, 4 h SD and recovery [Pre], cocaine/saline exposure, and a second 4 h SD and recovery [Post]. The timing of injections (indicated by red arrows) and SD (indicated by blue bar) relative to the light/dark cycle is given in the within day timeline. The light phase is indicated by the yellow box and the dark phase by the gray boxes.

Male C57BL/6 mice were obtained from Charles Rivers Laboratories and were housed under a 12/12 h light/dark cycle (22°C ± 1°C), with *ad libitum* access to food and water. Mice were implanted with EEG/EMG electrodes and following 14 days of recovery, acclimated to the recording environment which consisted of open-topped cages suspended ∼2 cm above the belt of a treadmill apparatus and a tether connection through a commutator which allows relatively free movement. During acclimation, mice were semi-randomized into an experimenter-administered model of binge cocaine (cocaine) and binge saline (saline) groups for two experimental designs; in design #1 animals experienced repeated experimenter-administered binge injections for 13 days followed by undisturbed recovery for 35 days, in design #2 animals underwent SD, followed by 13 days of experimenter-administered binge injections, followed by a second acute SD (Figure 1). Cocaine animals were run alongside saline animals (except for the first cohort which consisted of two cocaine animals) within a single design. Mice were singly housed due to the presence of the recording tether, but a gap between the cage bottom and belt surface allowed olfactory and some tactile interaction. All experiments were approved by the North Texas VA Health Care System IACUC and were accordance with recommendations in the Guide for Care and Use of Laboratory Animals (United States Research Council).

### Surgical Procedures

Mice were anesthetized with isoflurane (induction dose 3%, maintenance dose ∼1–2%) and kept on a heating pad. After the loss of tail pinch response, mice were placed into a stereotaxic apparatus, hair over the skull was sheared, and the skin cleaned and incised. Holes were drilled bilaterally over the frontal cortex (Bregma A/P + 1.7, M/L ± 1.77) and unilaterally over the parietal (Bregma A/P −1.7, M/L + 2.0) and occipital cortices (Bregma A/P 5.52, M/L −1.5) after which custom electroencephalograph (EEG) electrodes (coated wire with a female pin [Plastics One] soldered to small self-tapping screws were lowered). Commercial electromyograph (EMG) electrodes [Plastics One] were placed bilaterally in the nuchal muscle. Electrode pins were gathered into a plastic pedestal [Plastics One] and cemented to the skull. The skin was closed with an absorbable suture and the wound was covered with antibiotic ointment. Mice received buprenorphine for pain relief (2 injections) and were checked daily for 10 days following surgery. All mice recovered without complication.

### Design #1

To investigate the effect of chronic cocaine on long term sleep/waking activity animals underwent experimenter-administered binge cocaine followed by 5 weeks of forced abstinence. The time course of procedures is given in Figure 1. Mice underwent baseline, undisturbed recording for 4 days, 3x/d injections for 13 days, and forced abstinence for 35 days. This duration of abstinence was chosen since alterations in sleep behavior have been noted weeks into cocaine abstinence (Morgan et al., 2006; Chen et al., 2015). Daily injections consisted of 1 IP injection/h for 3 consecutive hours starting between ZT20–ZT21 such that the third injection was at least 1 h before ZT0 (lights on).

### Design #2

To investigate the effect of chronic cocaine on response to sleep deprivation animals underwent SD both before and after experimenter-administered binge cocaine exposure. The time course of procedures is given in Figure 1. Briefly, mice underwent baseline, undisturbed recording for 4 days, acute SD for 4 h with 68 h recovery, 3x/d injections for 13 days, and acute SD for 4 h with 68 h recovery. SD consisted of enforced waking from ZT0–ZT4. The same timing of injections was used as in design #1.

Mice in both groups were given soft chow (pellets moistened in water) after the last daily injection or similar times on baseline and forced abstinence days to reduce the anorexic effects of cocaine. Cocaine induced weight loss during the early part of the injection phase (group avg weight loss = 3.1 ± 0.4 g), resulting in a non-significant trend toward a difference in weight between groups {Two-Way ANOVA, Group effect #*p* = 0.052 [*F*(1, 21) = 4.253], Day effect *p < 0.0001 [*F*(4.294, 90.16) = 7.143], Group × Day effect *p = 0.0053 [*F*(4.294, 90.16) = 7.143], cocaine *n* = 12, saline *n* = 11}. This non-significant trend toward lower body weight continued into the post-injection period {Two-Way ANOVA, Group effect #*p* = 0.086 [*F*(1, 10) = 3.636], Day effect *p < 0.0001 [*F*(3.920, 39.20) = 53.16], Group × Day effect *p = 0.0031 [*F*(34, 340) = 1.867], *n* = 6/group}. Two mice in the cocaine group (both from design #2) did not complete the experiment; one died after the 1st injection on cocaine day 6 (necropsy indicated cocaine toxicity), the other was euthanized due to extreme lethargy after the second cocaine day (necropsy was unable to determine the cause of the lethargy).

### EEG/EMG Recordings

Mice were allowed to recover for at least 2 weeks following the end of surgical procedures after which they were given 1 week to acclimate to a recording tether plugged into a commutator which allows relatively free movement. A Grass Model 12LT amplifier system with PolyView Pro software [Grass Technologies/Natus Neurology] was used to acquire and filter (EEG 0.3–300, EMG 0.1–100) signals at a sampling rate of 256 Hz. Signals were extracted and scored for sleep/waking state using a custom Matlab-based sleepscorer module as described previously (Bjorness et al., 2016). States were scored in 10 s epochs into waking (low amplitude, mixed frequency EEG, modulated EMG), SWS (high amplitude, slow frequency EEG, slightly modulated EMG), and REM (low amplitude, fast frequency EEG, unmodulated EMG). Epochs with artifact were flagged and excluded from power analyses {% of epochs excluded to due presence of artifacts, cocaine = 2.03 ± 0.3, saline = 1.73 ± 0.18, not significantly different, Unpaired *T*-test *p* = 0.45; no difference in % epochs excluded due to the presence of artifacts across time, Mixed Model ANOVA with Tukey correction for multiple comparisons, Group effect not significant (n.s.) [*F*(1, 21) = 1.114], Time effect n.s. [*F*(51, 619) = 0.7943], Group × Time effect n.s. [*F*(51, 619) = 1.131]}. Power spectrum values were calculated in a 2 s window with 1 s overlap and a Hamming window using the pre-defined mean squared spectrum Matlab function. Slow wave activity (SWA; 0.5–4.5 Hz) was measured within SWS (SWS SWA) and within waking (W SWA). Slow wave energy (SWE) was calculated by summing SWA in all epochs during SWS (SWS SWE) or during waking (W SWE), while gamma energy (30–50 Hz) during waking was summed for discrete 15 min periods near the injection or weighing periods. The terminology of delta, synonymous with SWA, was used for delta/theta ratio, with theta power defined as the 5–10 Hz spectral power range.

### Sleep Deprivation

On SD days, the belt was started at ZT0 and stopped at ZT4 moving at a speed of ∼3 cm/s, the pace of a slow walk and considerably slower than speeds used for exercise (∼20 cm/s, Um et al., 2011). Mice were observed at multiple points during the SD period to ensure they were able to keep up with the belt. This duration and method of SD does not result in an increase in glucocorticoid-related genes (Bjorness et al., 2020) suggesting that a significant stress response would not be induced.

### Drugs

Cocaine hydrochloride (Sigma-Aldrich) was dissolved in a sterile saline. Animals in the cocaine groups received 15 mg/kg cocaine/IP injection. The dose, between-injection timing, and number of days repeated was based on a previously developed model of binge cocaine protocol designed to mimic human cocaine use patterns for rodent studies (Maisonneuve et al., 1995). Mice were weighed prior to the first daily injection; all injections were based on the daily weight and volumes per injection were < 0.1 mL.

### Outcome Measures

The outcome measures consisted of time in state parameters (sleep latency, time in state) and spectral power comparisons (SWE within SWS and W, SWA within SWS and W, and gamma energy prior to and following cocaine injections). Sleep latency was defined as the time between the injection (on injection days), end of the SD period (on SD days), or daily weighing (on post-injection days) and the entry into SWS (SL), entry into SWS of at least a 5 min duration (SL > 5 min), or entry into REM (REML). The SL > 5 min was used as an indication of stable sleep based on criteria used for SWS modeling (Franken et al., 2001; Bjorness et al., 2016). For daily SL, SL > 5 min, and REML, latency values following the three injections were averaged if there was sleep following either of the first two injections, otherwise was summed from the first injection to the first sleep that occurred following the third injection. Time in state was calculated based on the number of epochs per state in a 24 h period with state episodes designated to begin and end upon 3 consecutive 10 s epochs of a single state (Bjorness et al., 2016). Time in state was calculated as percent of the period or as normalized to the animal’s own baseline average time in state either for the entire day (24) or within a 4 h period (6, 4 h periods was used to capture time in state circadian distribution). SWE and SWA was normalized by state to the animal’s own baseline values either for the entire 24 h period or in 1 h bins using the same circadian time during baseline. SWE and SWA power values were binned in 1 h (time course within day) or 24 h (average for entire day). Gamma energy ratio relative to the pre-injection waking period (15 min of waking prior to the first injection on d1) was compared to the energy over the same waking duration following each injection (1, 2, 3) and following ZT13 on each injection day and was compared following weighing on each post-injection/forced abstinence day. The first 15 min of waking following injection or weighing was excluded to reduce the influence of direct handling on the gamma energy measure; gamma energy was expected to be remain elevated at 15 min post-cocaine since optogenetically-driven gamma entrainment is maximal 15 min after IP cocaine exposure (Dilgen et al., 2013). Fifteen minutes of waking beginning at least 15 min after the injection was not present following all the saline injections but did occur following most (∼62.5%). Gamma energy ratio relative to baseline (matched circadian time) was compared to energy over the same waking duration after the end of SD. Since animals were not handled following SD, the 15 min period started immediately after the belt was turned off. Delta/theta ratio was averaged in 4 h bins with 3 bins during the light phase and 3 bins during the dark phase. The experimental timeline was divided into separate phases for baseline, injection period (d1–13), early (p1–14) and late (p22–35) post-injection/forced abstinence period. Division of the post-injection/forced abstinence period was performed in order to include similar time periods as with the injection period and to be able to capture changes in behavior that may vary across abstinence as has been shown with outcomes such as cocaine craving (Grimm et al., 2001).

### Statistical Analysis

Data analysis was completed in Excel (Microsoft) and Prism (GraphPad). Statistical comparisons consist of Two-way Repeated Measures ANOVA (between phase or group), Mixed Model ANOVA (between phase or group when some values are missing, One Sample *T*-test (to compare vs. 100%), and Unpaired *T*-tests. For ANOVAs, multiple comparisons were run when there was a significant or trend toward significant effect; corrections for multiple comparisons were Sidak (2 points) or Tukey (>2 points). Values presented consist of average ± standard error unless otherwise noted; difference was considered significant at *p* ≤ 0.05 (non-significant) trends toward difference for *p* ≤ 0.1 are included. For all statistical comparisons *F* values (variable 1, variable 2, interaction) are provided, for significant or non-significant trends toward effects *p*-values are given, while *t* values and 95% Confidence Interval values of pairwise comparisons and One Sample comparisons are provided in Supplementary Tables 1–7. Non-significant effects are labeled as n.s. Two sets of repeated cocaine/saline include missing data; in one set (*n* = 1 cocaine, *n* = 2 saline, design #1) injection day 7 EEG/EMG data was not collected due to experimenter error, in another set (*n* = 2 cocaine, *n* = 2 saline, design #2) injection days 6–11 were not captured due to a computer malfunction. Additionally, one mouse (cocaine, design #2) damaged his tether during the 3rd forced abstinence week so his values for p21–35 are missing. Animals with missing injection days 6–11 were excluded from injection period analyses, while the presence of missing values of injection day 7 and p21–35 (*n* = 1) are noted in the figure legends are applicable. Animals from design 1 and 2 were combined during the injection period.

## Results

### Effect of Repeated Cocaine on Arousal

**FIGURE 2:**
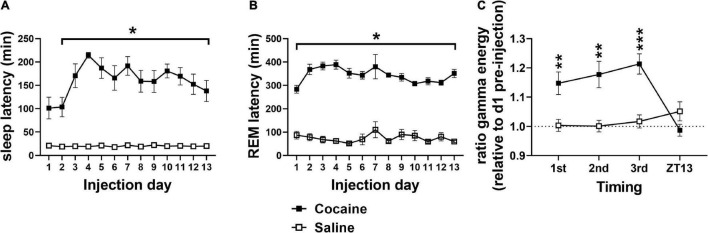
**(A)** Cocaine increased SL compared to saline with significant pairwise comparison effects on days 2–13 and a trend toward significance difference on day 1. **(B)** Cocaine increased REML compared to saline on all days. **(C)** Relative gamma energy (ratio to pre-injection waking gamma energy) increased following each of the three cocaine injections compared to saline, while gamma energy early in the dark phase (ZT13) was not significantly different between groups. Cocaine *n* = 10 [**(A,B)** one mouse missing value on day 7], saline *n* = 9 [**(A,B)** two mice missing values on day 7]. Asterisks indicate a significant difference between groups and lines indicate the days over which the group difference occurs. *P* and *F* values are given in Table 1 and statistics for all pairwise comparisons with significant difference or trends toward significance are given in Supplementary Table 1.

**FIGURE 3:**
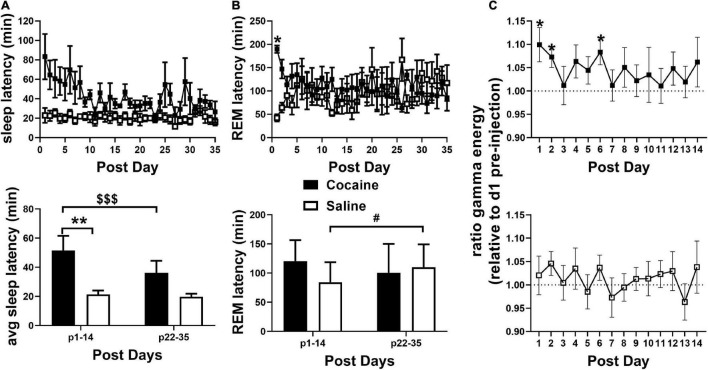
(**A**, top) SL remained elevated in the cocaine group compared to the saline group during the 35 days post-injection/forced abstinence period. (**A**, bottom) Average SL following handling/weighing remained higher in cocaine-experienced animals during the first 2 weeks compared to saline experienced animals throughout abstinence and compared to cocaine experienced animals in the latter portion of the post-injection/forced abstinence phase. (**B**, top) REML remained higher in cocaine-experienced animals on the first post-injection day only. Cocaine *n* = 6 (one mouse missing values days p21–35), saline *n* = 6. (**B**, bottom) Average REML did not vary between groups during the first two and last two post-injection/forced abstinence weeks, though saline-treated animals showed a non-significant trend toward increased REML across the post-injection/forced abstinence period, cocaine *n* = 5–6 (one mouse not included in p22–25 average), saline *n* = 6. **(C)** During the post-injection/forced abstinence phase relative gamma energy (ratio to pre-injection waking gamma energy) increased in the cocaine group (top) over several days of the first post-injection week, while waking gamma energy was unchanged in saline-experienced animals (bottom). Cocaine *n* = 5–6 (one mouse missing days 22–35), saline *n* = 6. Asterisks indicate a significant difference between groups **(A,B)** or from 1 on specific days [pre-injection gamma energy **(C)**], dollar signs and pound signs indicate a significant and non-significant trend toward a difference between time points with lines indicate the columns compared. *P* and *F* values are given in Table 1 and statistics for all pairwise comparisons with significant difference or trends toward significance are given in Supplementary Table 1.

Latency to fall asleep reflects a combination of sleepiness, circadian time, and physiological arousal (Bonnet and Arand, 2005). As expected of a psychostimulant, cocaine increased SL compared to saline (Figure 2A) with increases persisting into the post-injection phase (Figure 3A). Latency to enter REM was also increased following cocaine (Figure 2B) with increases persisting into the first day of the post-injection phase (Figure 3B). The difference in SL vs. SL > 5 min was significantly longer in cocaine treated than saline treated animals during the injection period (SL > 5 min—SL; cocaine = 64.2 ± 10.9, saline = 6.16 ± 0.96, Unpaired *T*-test, *p < 0.0001, cocaine *n* = 10, saline *n* = 9), but did not persist within the post-injection period [cocaine p1–14 = 14.81 ± 4.88, p22–35 = 12.11 ± 3.92, saline p1–14 = 10.85 ± 4.66, p22–35 = 12.52 ± 2.87, Mixed effects ANOVA with Sidak correction for multiple comparisons, Group effect n.s. *F*(1, 10) = 0.2837, Time effect n.s. *F*(1, 9) = 0.1464, Group × Time effect n.s. *F*(1, 9) = 0.2370, cocaine *n* = 5–6 (one mouse missing p22–35), saline *n* = 6]. In general, animals receiving saline achieved sleep, stable sleep, and REM sleep between within-day injections, while the animals receiving cocaine achieved little sleep, very little stable sleep, and no REM sleep between within-day injections [SL and REML (Supplementary Figures 1A,B); SL > 5 cocaine = 59.65 ± 0.16, saline = 21.56 ± 0.95 min]. In addition to the increased SL measures, arousal following each cocaine injection was observed by an increase in waking gamma spectral energy (Figure 2C), high frequency EEG activity associated with behavioral arousal (Maloney et al., 1997), with increased gamma energy following weighing persisting briefly into the post-injection/forced abstinence period (Figure 3C). Overall, these results indicate that cocaine acutely increases arousal, as expected, and further suggests that arousal in response to external stimuli such as handling, persists into the post-injection/forced abstinence period. Further, the increase in sleep latency during abstinence from cocaine is consistent with a previous report from a small population of cocaine-dependent men (Johanson et al., 1999).

### Effect of Repeated Cocaine on State Time and Transitions

**FIGURE 4:**
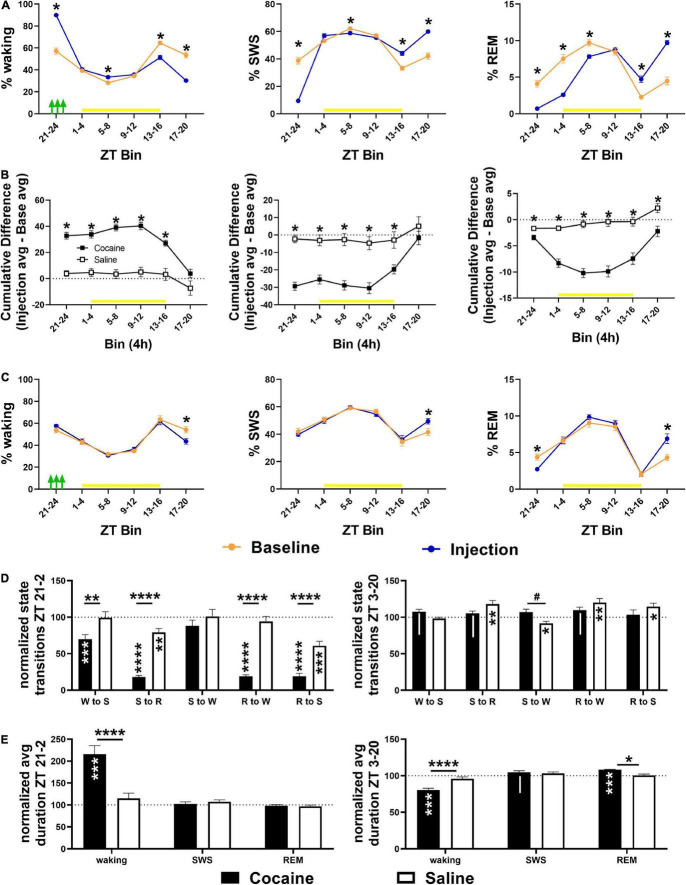
**(A)** Compared to baseline, cocaine increased waking and decreased SWS and REM during the injection period (indicated by green arrows) followed by a decrease in waking/increase in sleep during the early and middle part of the dark phase with more waking/less SWS during the middle and end of the light phase during the late vs. early injection stage. **(B)** The cumulative difference between baseline and injection days (Average injection—average baseline) was significantly different between cocaine and saline-treated groups across states, though only the% REM remained significantly different across the 24 h. **(C)** Compared to baseline, saline decreased waking time and increased SWS time during the middle of the dark phase while REM time was decreased during the injection bin with rebound increase during the middle of the dark phase. Cocaine *n* = 10, saline *n* = 9. Asterisks indicate a significant difference within (left, right columns) or between (middle column) groups for specific 4 h bins. The yellow bar indicates the light phase. (**D**, top) Transitions between states decreased in cocaine-treated animals during the cocaine exposure period and early part of the light phase (6 h encompassing last 4 h of the dark phase and first 2 h of the light phase) with the exception of SWS to waking transitions that remained similar to baseline levels and to saline (cocaine *n* = 10, saline *n* = 9). (**D**, bottom) During the remaining portion of the 24 h period (ZT3–ZT20), transitions in cocaine-exposed animals were non-significantly different from baseline (waking to SWS, SWS to REM, REM to waking) and saline (SWS to waking), while state transitions in saline-exposed animals (SWS to REM, SWS to waking, REM to waking, REM to SWS) were significantly different from baseline. (**E**, top) Waking duration significantly increased in cocaine-exposed during the cocaine exposure period and early part of the light phase (ZT 21–24, ZT 0–2) compared to baseline and compared to saline (cocaine *n* = 10, saline *n* = 9). (**E**, bottom) During the remaining period of the 24 h period (ZT 3–20), waking was significantly shorter in cocaine-exposed animals compared to baseline and saline, while REM was significantly longer, and SWS was non-significantly longer compared to baseline. The dashed line at 100 indicates baseline level. Asterisks and pound symbols above lines indicate a significant and non-significant trend toward a difference between groups, respectively, at those states, asterisks and pipes within bars indicate a within-group significant and non-significant trend toward a difference from baseline, respectively. *P* and *F* values and statistics for all pairwise comparisons with significant difference or trends toward significance are given in Supplementary Table 2.

Cocaine induced a high percentage of waking during the 4 h period in which three cocaine injections were given, followed by a delayed decrease in waking during the early and middle portion of the dark phase (beginning 12 h after the injection bin; Figure 4A), but resulting in no overall cumulative difference in waking between average injection days and average baseline days relative to saline exposure (Figure 4B). This pattern, in the opposite direction, was observed for SWS, while REM sleep was decreased for multiple consecutive 4 h bins prior to increasing during the dark phase (Figure 4A) resulting in a net decrease in cumulative difference relative to saline exposed animals (Figure 4B) due to a non-significant decrease and significant increase in REM compared to baseline in cocaine and saline-treated animals, respectively (data not shown, One sample *T*-test compared to 100, cocaine = 94.48 ± 2.59%, #*p* = 0.062, *n* = 10, saline = 107.2 ± 2.87%, *p = 0.037, *n* = 9). Saline decreased waking and increased SWS compared to baseline during the middle of the dark phase (∼20 h after the injections) despite no change in waking or SWS in other bins, while REM decreased during the injection period relative to baseline with rebound increases during the middle of the dark phase (Figure 4C). A heat map of the percent time in state in 4 h bins across each baseline and injection day is given to show the timing of the comparatively strong wake promotion/sleep inhibition influence of cocaine with delayed recovery during each injection day and the relative similarity of time in state across baseline days and across saline injection days (Supplementary Figures 2A,B). The decrease in waking during the middle of the dark phase following cocaine persisted throughout the post-injection/forced abstinence period (Supplementary Figure 3A), while the increase in REM sleep in saline exposed animals persisted in the post-injection/forced abstinence period, shifting earlier within the dark phase (Supplementary Figure 3D) with no cumulative difference in any state between groups (Supplementary Figures 3B,C).

Cocaine reduced state transitions during the end of the dark phase and early portion of the light phase compared to baseline and to saline, while saline reduced transitions between sleep states during this period compared to baseline (Figure 4D). During the remaining period of the light phase and early and mid-dark phase, differences in state transitions between cocaine and baseline or saline only reached the level of non-significant trends, while saline increased state transitions to and from REM sleep and decreased transitions from SWS to waking (Figure 4D). The decrease in transitions between states following cocaine exposure was driven by a large increase in average waking duration, while waking duration decreased and REM duration increased compared to baseline and saline during the remaining light phase and early-to-mid dark phase (Figure 4E).

### Effect of Repeated Cocaine on Spectral Power

**FIGURE 5:**
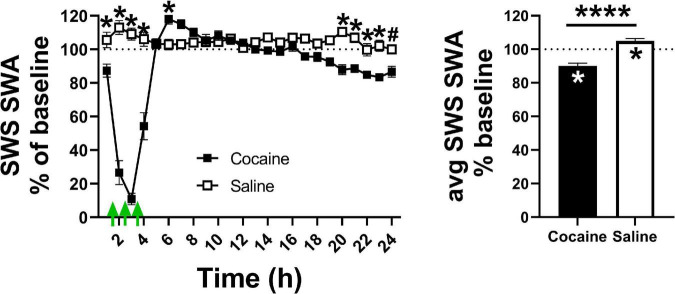
(Left) Cocaine exposure decreased sleep intensity, as measured by SWS SWA, during the hours in which cocaine was administered (indicated by the green arrows) after which sleep intensity briefly rebounded then ended below saline. (Right) Cocaine induced a net decrease in average SWS SWA compared to saline and baseline levels while saline induced a net increase in SWS SWA compared to baseline levels. Cocaine *n* = 10, saline *n* = 9. The dashed line at 100 indicates baseline level, the asterisks above the symbols or lines indicate a between group difference at those timepoints, the asterisks within bars indicate a within-group difference from baseline. *P* and *F* values are given in Table 1 and statistics for all pairwise comparisons with significant difference or trends toward significance are given in Supplementary Table 3.

SWS SWA, a measure of sleep intensity (Borbely and Neuhaus, 1979), sharply decreased during the cocaine portion of the 24 h period before briefly rebounding above saline levels and then dipping below saline levels toward the latter portion of the 24 h period (Figure 5, left). Saline increased SWS SWA during the injection portion of the 24 h period. Across the 24 h period, cocaine induced a net decrease in SWS SWA compared to baseline, while saline induced a modest, but significant net increase in SWS SWA compared to baseline (Figure 5, right). The pattern of decreasing SWS SWA during the end of the dark phase remained evident in cocaine-treated animals during the early portion of the post-injection/forced abstinence period, though power values were not significantly different than saline experienced animals, possibly due to high within-group variability (Supplementary Figure 4A). The pattern of decreased SWS SWA was no longer apparent during the latter portion of the post-injection/forced abstinence phase (Supplementary Figure 4B). There was no significant difference between groups or relative to baseline at either portion of the post-injection/forced abstinence phase (Supplementary Figure 4C). Overall, these results suggest that sleep achieved in the presence of cocaine is less intense than typically occurs at matched circadian timepoints, that sleep lost followed cocaine exposure is sufficient to induce a homeostatic response as determined by the rebound (i.e., increased intensity as compared to typical intensity at matched circadian time points), that this rebound is insufficient to fully recover SWS SWA within the remaining ∼20 h period, that saline exposure is sufficient to modestly increase sleep intensity despite a lack of sleep loss during the injection period, and that, in some animals, changes in sleep intensity within specific portions of the circadian period may persist after the end of daily cocaine exposure.

**TABLE 1:** Table of *P* and *F* values for all main comparisons.

Mixed effects ANOVA	*F*(DFn, DFd)	*P*-value
**SL Injection Period, Figure 2A**		
Day*	*F*(3.768, 63.12) = 2.718	0.0403
Group***	*F*(1, 17) = 131.0	< 0.0001
Day × Group**	*F*(12, 201) = 2.685	0.0023
**REML injection period, Figure 2B**		
Day	*F*(3.702, 62.00) = 1.651	0.1769
Group***	*F*(1, 17) = 723.5	< 0.0001
Day × Group*	*F*(12, 201) = 2.183	0.0138
**Avg waking gamma energy ratio, Figure 2C**		
Day***	*F*(3, 30) = 9.525	0.0001
Group*	*F*(1, 10) = 8.426	0.0158
Day × Group***	*F*(3, 18) = 33.20	< 0.0001
**SL post period, Figure 3A**		
Day#	*F*(3.479, 33.26) = 2.251	0.0924
Group*	*F*(1, 10) = 8.066	0.0175
Day × Group*	*F*(34, 325) = 1.607	0.0203
**Avg SL post period, Figure 3A**		
Day**	*F*(1, 9) = 11.43	0.0081
Group*	*F*(1, 10) = 6.924	0.0251
Day × Group*	*F*(1, 9) = 7.789	0.021
**REML post period, Figure 3B**		
Day	*F*(4.719, 45.11) = 0.4603	0.7937
Group	*F*(1, 10) = 0.2822	0.6069
Day × Group***	*F*(34, 325) = 1.726	0.009
**Avg REML post period, Figure 3B**		
Day	*F*(1, 9) = 0.08364	0.779
Group	*F*(1, 10) = 0.3385	0.5736
Day × Group*	*F*(*F*(1, 9) = 10.38	0.0105
**SWS SWE% baseline_Figure 6A (top)**		
*Day	*F*(3.655, 61.23) = 2.989	0.0291
***Group	*F*(1, 17) = 19.68	0.0004
***Day × Group	*F*(12, 201) = 4.124	< 0.0001
**SWS% baseline_Figure 6A (upper middle)**		
Day*	*F*(4.828, 80.87) = 2.953	0.018
Group	*F*(1, 17) = 1.389	0.2549
Day × Group***	*F*(12, 201) = 4.145	< 0.0001
**SWS SWA% baseline_Figure 6A (lower middle)**		
Day	*F*(5.537, 92.74) = 1.708	0.1335
Group***	*F*(1, 17) = 49.96	< 0.0001
Day × Group	*F*(12, 201) = 1.319	0.2095
**W SWE% baseline_Figure 6B (top)**		
Day***	*F*(6.752, 113.1) = 5.121	< 0.0001
Group	*F*(1, 17) = 2.563	0.1278
Day × Group***	*F*(12, 201) = 5.801	< 0.0001
**W% baseline_Figure 6B (upper middle)**		
Day*	*F*(5.973, 100.0) = 2.374	0.0349
Group#	*F*(1, 17) = 3.485	0.0793
Day × Group***	*F*(12, 201) = 3.511	< 0.0001
**W SWA % baseline_Figure 6B (lower middle)**		
Day***	*F*(4.656, 77.99) = 7.004	< 0.0001
Group	*F*(1, 17) = 1.977	0.1777
Day × Group**	*F*(12, 201) = 2.506	0.0044

**One sample *t*-test**	***t*, df**	***P*-value (two tailed)**

**Avg waking gamma energy ratio, Figure 3C**		
Cocaine		
Day 1*	*t* = 2.705, df = 5	0.0425
Day 2*	*t* = 3.259, df = 5	0.0225
Day 6*	*t* = 3.038, df = 5	0.0288
**% avg SWS SWA across the 24 h period, Figure 5**		
Cocaine	*t* = 6.532, df = 9	0.0001
Saline	*t* = 3.482, df = 8	0.0083
**SWS metrics, Figure 6 (bottom)**		
SWS SWA_cocaine	*t* = 6.532, df = 9	0.0001
SWS SWA_saline	*t* = 3.482, df = 8	0.0083
SWS SWE_saline	*t* = 4.362, df = 8	0.0024
**W metrics, Figure 6 (bottom)**		
W SWA_cocaine	*t* = 3.647, df = 10	0.0045
W SWA_saline	*t* = 3.097, df = 7	0.0174
W_cocaine	*t* = 1.239, df = 9	0.2466
W_saline	*t* = 1.365, df = 8	0.2095
W SWE_cocaine	*t* = 3.091, df = 9	0.0129

**Two way ANOVA**	***F*(DFn, DFd)**	***P*-value**

**% waking in 4 h bins_cocaine, Figure 4A**		
ZT bin***	*F*(3.106, 27.95) = 169.6	< 0.0001
Condition	*F*(1.000, 9.000) = 1.449	0.2595
ZT bin × Condition***	*F*(3.136, 28.22) = 77.20	< 0.0001
**% SWS in 4 h bins_cocaine, Figure 4A**		
ZT bin***	*F*(3.008, 27.07) = 176.2	< 0.0001
Condition	*F*(1.000, 9.000) = 0.1987	0.6663
ZT bin × Condition***	*F*(3.063, 27.57) = 82.81	< 0.0001
**% REM in 4 h bins_cocaine, Figure 4A**		
ZT bin***	*F*(3.573, 32.16) = 100.2	< 0.0001
Condition#	*F*(1.000, 9.000) = 5.010	0.052
ZT bin × Condition***	*F*(2.707, 24.36) = 58.47	< 0.0001
**Cumulative difference % waking in 4 h bins_cocaine, Figure 4B**		
ZT bin***	*F*(2.582, 43.89) = 48.56	< 0.0001
Group***	*F*(1, 17) = 46.06	< 0.0001
ZT bin × Group***	*F*(5, 85) = 12.33	< 0.0001
**Cumulative difference % NREM in 4 h bins_cocaine, Figure 4B**		
ZT bin***	*F*(2.216, 37.68) = 39.33	< 0.0001
Group***	*F*(1, 17) = 23.25	0.0002
ZT bin × Group***	*F*(5, 85) = 12.06	< 0.0001
**Cumulative difference % REM in 4 h bins_cocaine, Figure 4B**		
ZT bin***	*F*(2.296, 39.03) = 31.98	< 0.0001
Group***	*F*(1, 17) = 46.20	< 0.0001
ZT bin × Group***	*F*(5, 85) = 16.04	< 0.0001
**% waking in 4 h bins_saline, Figure 4C**		
ZT bin***	*F*(2.882, 23.05) = 43.79	< 0.0001
Condition	*F*(1.000, 8.000) = 1.798	0.2168
ZT bin × Condition***	*F*(2.923, 23.38) = 9.052	0.0004
**% SWS in 4 h bins_saline, Figure 4C**		
ZT bin***	*F*(2.916, 23.32) = 34.08	< 0.0001
Condition	*F*(1.000, 8.000) = 0.8895	0.3732
ZT bin × Condition***	*F*(3.119, 24.95) = 7.380	0.0009

**Two way ANOVA**	***F*(DFn, DFd)**	***P*-value**

**% REM in 4 h bins_saline, Figure 4C**		
ZT bin***	*F*(2.916, 23.32) = 34.08	< 0.0001
Condition	*F*(1.000, 8.000) = 0.8895	0.3732
ZT bin × Condition***	*F*(3.119, 24.95) = 7.380	0.0009
**State transitions ZT 21–2, Figure 4D**		
Transition***	*F*(4, 85) = 28.41	0.0001
Group***	*F*(1, 85) = 123.9	0.0001
Transition × Group***	*F*(4, 85) = 7.830	0.0001
**State transitions ZT 3–20, Figure 4D**		
Transition**	*F*(4, 80) = 4.152	0.0042
Group	*F*(1, 80) = 0.5296	0.4689
Transition × Group**	*F*(4, 80) = 4.358	0.0031
**Episode duration ZT 3–20, Figure 4E**		
State***	*F*(2, 51) = 27.39	< 0.0001
Group***	*F*(1, 51) = 15.48	0.0003
State × Group***	*F*(2, 51) = 17.17	0.0001
**Episode duration ZT 21-2, Figure 4E**		
State***	*F*(2, 51) = 41.10	< 0.0001
Group	*F*(1, 51) = 1.681	0.2007
State × Group***	*F*(2, 51) = 17.53	< 0.0001
**% SWS SWA across the 24 h period, Figure 5**		
Time***	*F*(23, 391) = 37.52	< 0.0001
Condition	*F*(1, 17) = 50.41	< 0.0001
Time × Condition***	*F*(23, 391) = 48.73	< 0.0001
**% SWS SWA_4 h SD with 24 h recov_cocaine, Figure 7A (top)**		
Time***	*F*(27, 135) = 73.53	< 0.0001
Condition	*F*(1, 5) = 0.2165	0.6613
Time × Condition***	*F*(27, 135) = 2.797	< 0.0001
**% SWS SWA_4 h SD with 24 h recov_saline, Figure 7A (middle)**		
Time***	*F*(27, 108) = 77.55	< 0.0001
Condition	*F*(1, 4) = 1.443	0.296
Time × Condition#	*F*(27, 108) = 1.458	0.0901
**% SWS SWA_4 h SD with 24 h recov_cocaine and saline, Figure 7A (bottom)**		
Time***	*F*(3.751, 33.76) = 58.83	< 0.0001
Condition	*F*(1, 9) = 0.4548	0.517
Time × Condition***	*F*(27, 243) = 3.124	< 0.0001
**% SWS_4 h SD with 24 h recov_cocaine, Figure 7B (top)**		
Time***	*F*(27, 135) = 12.73	< 0.0001
Condition	*F*(1, 5) = 0.03302	0.8629
Time × Condition***	*F*(27, 135) = 3.001	< 0.0001
**% SWS_4 h SD with 24 h recov_saline, Figure 7A (middle)**		
Time***	*F*(27, 108) = 7.818	< 0.0001
Condition	*F*(1, 4) = 2.187	0.2133
Time × Condition	*F*(27, 108) = 0.6843	0.8713
**% SWS_4 h SD with 24 h recov_cocaine and saline, Figure 7A (bottom)**		
Time***	*F*(3.185, 28.67) = 11.59	< 0.0001
Condition	*F*(1, 9) = 1.640	0.2324
Time × Condition#	*F*(27, 243) = 1.404	0.0951

**Unpaired *t*-test (Two-tailed)**	***t*, df**	***P*-value**

**% avg SWS SWA across the 24 h period, Figure 5**		
	*t* = 7.100, df = 17	< 0.0001

Symbols indicate significance level, *p < 0.05, **p < 0.01, ***p < 0.001, ^#^p < 0.1.

**FIGURE 6:**
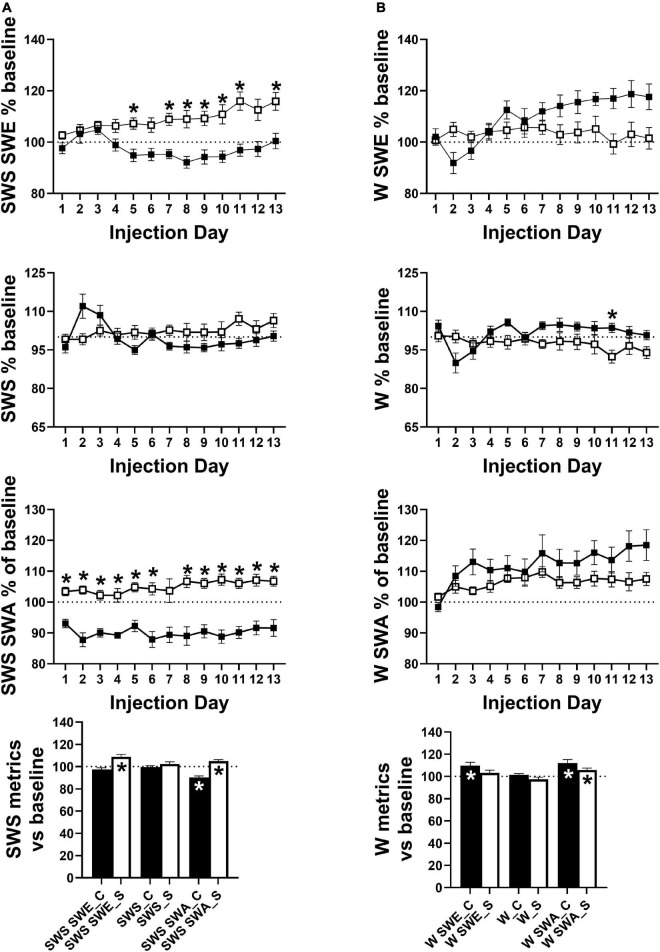
**(A)** SWS SWE was significantly lower in cocaine-treated animals compared to saline-treated animals driven not by a difference in SWS time but driven by lower SWS SWA. The group difference in SWS SWE and SWS SWA resulted from differences in SWS SWA relative to baseline with decreased and increased average SWS SWA in cocaine-treated and saline-treated animals, respectively. **(B)** W SWE, W time, and W SWA did not differ significantly by group, however, W time was non-significantly higher in cocaine-treated animals while all metrics showed an effect of time and interaction between group and time with W SWE and W SWA diverging by group as the 13 days period progressed. While both groups showed increased average W SWA relative to baseline, average W SWE was only increased above baseline in cocaine-treated animals. Cocaine *n* = 10, saline *n* = 9. Dashed lines at 100 indicates baseline level, asterisks above symbols indicate group difference at those timepoints, asterisks within bars indicate a within-group difference from baseline. *P* and *F* values are given in Table 1 and statistics for all pairwise comparisons with significant difference or trends toward significance are given in Supplementary Table 4.

Across the 24 h period, total SWS SWE became significantly lower in cocaine-exposed animals compared to saline-exposed animals across the 13 days injection period, due predominantly to a decrease in sleep intensity (i.e., SWS SWA) across the majority of the 13 days injection period alongside non-significantly lower SWS time that emerged during the latter portion of the 13 days period (Figure 6A). Averaging across the 13 days period, SWS SWA was decreased by cocaine and increased by saline, consistent with the changes observed in the average across the 24 h period (discussed above, Figure 5 right), while net SWS SWE increased in response to saline as compared to baseline (Figure 6A). Though not significantly different between groups, SWE during W (W SWE) was significantly elevated above baseline values in cocaine-exposed animals, likely due to an elevation in W SWA above baseline values and a non-significant trend toward increased waking time across the 24 h period (Figure 6B). Saline exposed animals also showed a small but significant increase in W SWA relative to baseline. W SWA occurs in rodents when stationary (Schultheiss et al., 2020), increases following SD (Franken et al., 2001; Vyazovskiy et al., 2011), and is hypothesized to be an indicator of sleep pressure (Cajochen et al., 2001; Tinguely et al., 2006); while the cocaine-induced increase in W SWA may be attributed to the significant increase in W during the end of the dark phase which may result in increased sleep pressure, the saline-induced increase in W SWA is unlikely to be caused by a change in sleep pressure.

During the post-injection/forced abstinence phase, avg SWS SWA returned to baseline levels in both groups with no differences between groups or across time, while SWS SWE remained elevated during the early portion of the post-injection/forced abstinence phase in saline experienced animals and an increase in SWS time emerged during the latter portion of the post-injection/forced abstinence phase, matched by a decrease in W time in saline experienced animals (Supplementary Figure 4D). W SWA remained elevated above baseline values during the early portion of the post-injection/forced abstinence phase in cocaine-experienced animals and throughout the post-injection/forced abstinence phase in saline-experienced animals, while W SWE was significantly and non-significantly elevated above baseline in the early portion of the post-injection/forced abstinence phase in saline and cocaine-experienced animals, respectively (Supplementary Figure 4E). The resolution of increased W SWA across the post-injection/forced abstinence phase in cocaine-experienced animals is consistent with the return of SWS SWA intensity across the 24 h period to baseline levels. The persistence of increased W SWA across the post-injection/forced abstinence period alongside the decrease in W during the latter portion of the same period in saline-experienced may indicate a decrease in activity during waking in this group. Furthermore, while W SWA across post-injection/forced abstinence days did not differ between groups, an interaction between group and time was observed with W SWA appearing to begin to diverge between groups around post week 3 (Supplementary Figure 4E) suggesting that waking locomotor activity may decrease across the post-injection/forced abstinence period in saline-experienced animals. Locomotor activity was not directly measured so possible changes in activity during waking remain speculative; however, delta/theta was significantly increased above baseline in all 4 h bins and increased from early to late post-injection/forced abstinence in saline-experienced animals while cocaine-experienced animals showed increased delta/theta during a subset of the 4 h bins with no change across time (Supplementary Figure 5A). Additionally, waking episode duration was significantly reduced during the early portion of the dark phase in saline-experienced animals compared to baseline with a trend toward increased waking episode duration during the end of the dark phase (Supplementary Figure 5B). The increase in delta/theta indicates that increased W SWA occurred without a matching increase in theta activity which supports the speculation of reduced locomotor activity during waking given the known relation between theta activity and locomotor activity (Vanderwolf, 1975), while the shift in the timing of consolidated waking may be related to the presence of soft chow which was given after daily weighing during the end of the dark phase. Eating soft chow during the end of the dark phase may have reduced pellet food consumption during the early part of the dark phase when long waking episodes typically occur in mice.

### Effect of Repeated Cocaine Experience on Response to Sleep Deprivation

**FIGURE 7:**
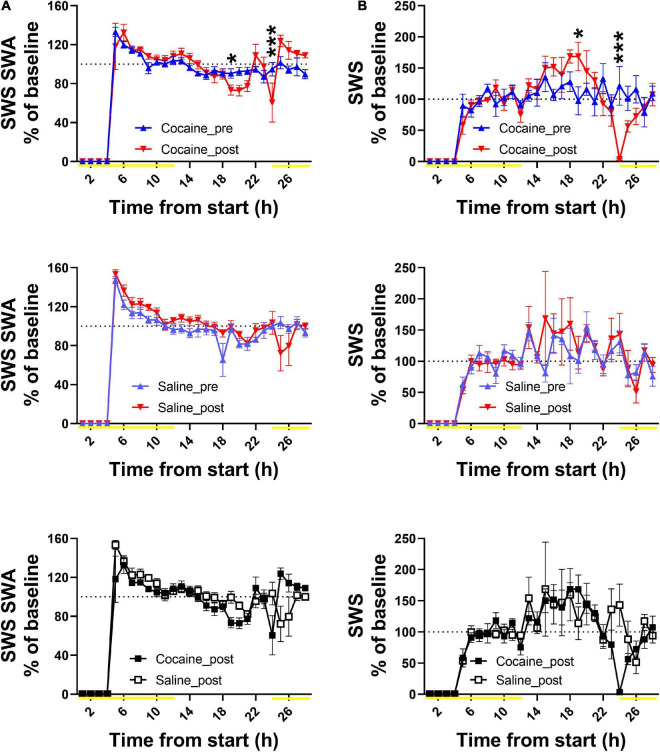
Both cocaine- and saline-treated animals showed similar SWS SWA **(A)** and SWS time **(B)** response to a 4 h sleep deprivation challenge. The time course of SWS SWA% baseline and SWS time% baseline was modified by cocaine exposure as compared to response to the same 4 h SD challenge prior to the initial cocaine exposure. Saline induced a non-significant trend toward a change in the time course of SWS SWA% baseline while SWS time% baseline was unchanged compared to the response prior to the initial saline exposure. The cocaine-induced change in SWS SWA% baseline time course was also observed when comparing between cocaine- and saline-treated animals, while the change in SWS time% baseline became a non-significant trend toward a difference. Note the post data in panels **(A,B)** top and middle graphs is replotted in the bottom between-groups comparison graph. Data was plotted for 28 h to include the 4 h SD period followed by response over the next 24 h. Yellow lines indicate light phase, dashed lines at 100 indicate baseline levels, and asterisks above symbols indicate difference between conditions at those specific timepoints. Cocaine *n* = 6, saline *n* = 5. *P* and *F* values are given in Table 1 and statistics for all pairwise comparisons with significant difference or trends toward significance are given in Supplementary Table 7.

Cocaine influenced the time course but not magnitude of homeostatic response to SD as determined by SWS SWA with similar effects in the SWS time, while saline induced a non-significant trend toward a change in the time course of SWS SWA which was absent in SWS time (Figures 7A,B). In both groups, SWS SWA and SWS time was absent during the SD period, followed by a rebound (increase above baseline) in SWS SWA immediately following SD and a delayed rebound in SWS time. There was no significant difference in SWS SWA or SWS time response to SD between groups, though there was a difference in the time course of SWS SWA response (Figures 7A,B). SWS SWE as a percent of baseline was also unchanged between groups and between conditions (prior to and following cocaine/saline exposure {data not shown, Two Way ANOVA with Sidak correction Group effect n.s. [*F*(1, 9) = 1.118], Time effect n.s. [*F*(1, 9) = 2.750], Group × Time effect n.s. [*F*(1, 9) = 2.177], cocaine *n* = 6, saline *n* = 5}. Overall, in contrast to the reduced average SWS SWA during the cocaine exposure days (as discussed above, Figure 5), homeostatic response to an externally enforced extended waking challenge was maintained following the end of cocaine exposure. Additionally, cocaine experience did not alter arousal during SD as determined by the lack of difference in SL (Supplementary Figures 6A–C) and gamma energy measures (Supplementary Figure 6D) between groups and across conditions.).

## Discussion

Increased arousal, indicated by increased SL, increased relative gamma ratio, and reduced sleep during the end of the dark phase was increased throughout the 13 days cocaine/saline administration period, with increases in SL and relative gamma ratio persisting days (gamma) to weeks (SL) into the post-injection/forced abstinence phase. Since homeostatic responses to SD were unchanged, plastic alterations of the arousal system rather than altered sleep need may account for these observations.

One arousal-related area known to undergo plastic changes following cocaine exposure is the orexin/hypocretin system. Self-administration of cocaine in an intermittent pattern increases activity of orexin neurons during abstinence when the animals are returned to the environment in which cocaine was received (James et al., 2019). Furthermore, both self-administered and passive cocaine increases mEPSC frequency indicative of presynaptic plasticity of orexin neurons (Yeoh et al., 2012) and repeated passive cocaine exposure (as occurred with our repeated cocaine protocol) increases experience-dependent potentiation of excitatory synapses on orexin neurons, an effect that persists into withdrawal (Rao et al., 2013). Together these data support a role for persistently altered orexin system excitability in the observed post- cocaine arousal.

The increase in arousal occurred at the end of the active phase and was followed by a decrease in arousal (as indicated by time in waking) at the beginning of the active phase suggesting that there was not an overall increase in arousal across the 24 h period, but rather a shift in the distribution of arousal (Figure 4 and Supplementary Figure 3). The reductions in waking during the middle of the dark phase during the cocaine phase and throughout the post-injection/forced abstinence phase may be akin to excessive daytime sleepiness reported in humans actively using cocaine and during early abstinence (Johanson et al., 1999). If the increase in arousal following cocaine is driven by changes in orexin activity, as speculated above, it may suggest that repeated administration of cocaine within a specific circadian range shifts the timing of orexin neuronal activity as occurs with availability of food under restricted feeding conditions (Jimenez et al., 2013). It is unknown whether animals anticipated daily procedures since locomotor activity, vocalizations, and temperature, which have been used to infer cocaine anticipation (Ma et al., 2010; Jansen et al., 2012), were not measured here; however, there was no clear indication of anticipation in terms of the animals consistently waking prior to the injection or post-injection weighing.

An increase in W SWA in the cocaine group was expected due to increased sleep pressure (Cajochen et al., 2001; Tinguely et al., 2006) since SWA intrudes into waking during SD (Franken et al., 2001; Vyazovskiy et al., 2011). Additionally, the increase in W SWA may be due to electrophysiological changes in thalamocortical neurons that underlie increases in SWA following acute 3x within-day cocaine (Urbano et al., 2009). The cause of increased W SWA in saline-treated animals is less clear but may be due to reduced activity during waking (Schultheiss et al., 2020). Animals were given soft chow daily to mitigate potential weight loss in cocaine-treated animals since cocaine suppresses food intake (Wolgin and Hertz, 1995). While food consumption was not measured, soft chow was generally ignored by all mice over the first few days of the experiment after which saline-treated animals were observed eating the soft chow within minutes of daily presentation. Thus, availability of soft chow may have altered the pattern of food intake, particularly in the saline control group. Though speculative, a change in food intake is consistent with the reduction in waking episode duration and increase in delta/theta ratio, an indirect indicator of activity in rodents (Li et al., 2012), during the early part of the dark phase under post-injection/forced abstinence conditions that was observed in saline-treated animals.

In contrast to the reduction in sleep homeostasis following ethanol exposure and withdrawal (Thakkar et al., 2015), repeated 3x/d cocaine does not alter homeostatic response to a SD challenge despite sleep intensity being reduced during daily cocaine exposure. This is consistent with a lack of change in sleep efficiency (time asleep relative to time in bed) following SD in humans with cocaine use disorder (Trksak et al., 2013).

Reduced SWS-SWA observed during cocaine administration could be an indication of reduced sleep homeostatic response, however, the characteristic physiological SWS SWA response may be attenuated by either or both of two factors. First, the SWA is likely to be masked by increased arousal induced by acute cocaine stimulant effects. Second, increased W SWA can alleviate sleep pressure caused by prolonged arousal (Franken et al., 2001) as occurred with the cocaine protocol.

### Limitations

While we believe that these experiments provide two important additions to the literature, the daily measurement of arousal throughout 13 days of repeated 3x/d cocaine and 5 weeks of forced abstinence and the test of homeostatic response to SD using spectral power, there were several notable limitations. First, the group size was relatively modest outside the injection phase so confidence in our interpretation of chronic cocaine’s effect on SD response and recovery across forced abstinence would be increased if independently replicated; *F* and *T* values from all statistical analyses were provided to aid comparison with future reports. Individual differences in response to cocaine (for example, locomotor activity, Gulley et al., 2003; reinforcement, Belin et al., 2011; Perry et al., 2013) suggest that there may be subsets of animals whose sleep is differentially susceptible/resilient to cocaine exposure or repeated IP injection for which larger data sets may allow groups to be divided into susceptible and resilient cohorts. Second, these experiments exclusively featured males so an important future step would be to repeat in females. Estrous phase can influence both sleep (Koehl et al., 2003) and cocaine (Kerstetter et al., 2008)–related behaviors, while cocaine can, in turn, influence estrous phase (King et al., 1993) so sex differences may exist. Use of a single sex was based on the limited availability of equipment and males were chosen for simplicity of design since estrous phase could be another source of intragroup variability. Third, a single cocaine protocol was used so it is unclear how alternate exposures (higher/lower dose, more/fewer repeats, intermittent instead of daily, distributed across the 24 h period instead of clustered across 3 h) would impact sleep-related outcomes; an important consideration given the highly variable nature of cocaine use in humans and one that would be expected to influence development of dependence. The protocol used here (3x/d for 13 days) was developed with the intent of mimicking human use patterns and is sufficient to decrease basal dopamine tone (Maisonneuve et al., 1995). Relatedly, cocaine exposure occurred during a single circadian phase so the potential influence of the circadian rhythm on sleep-related outcomes is unknown. Cocaine influences expression of several clock genes (Falcon et al., 2013), while circadian rhythm broadly influences cocaine-related behaviors (Webb et al., 2015) adding, as with hormonal cycles, complications for experimental design and data interpretation. Fourth, the presence of soft chow could influence timing of food intake, necessarily influencing timing of waking (and therefore sleep) and confounding interpretation of sleep timing changes. Inclusion of an additional control group that does not receive soft chow or use of a supplemental diet such as Ensure would be beneficial to control for or avoid possible effects of soft chow. Fifth, forced abstinence may induce a withdrawal syndrome as has been inferred in rodents by various anxiety-anhedonia measures, including vocalization (Covington and Miczek, 2003), marble burying (Basso et al., 1999), intracranial self-stimulation (Stoker and Markou, 2011), and glucose/sucrose intake (Carroll and Lac, 1987). While withdrawal from cocaine is often considered milder than withdrawal from other drugs of abuse due to the general lack of physical symptoms, negative physiological states such as anxiety or anhedonia could influence sleep (Horvath et al., 2016; Barthel et al., 2020) or the response to SD (Minkel et al., 2012; Goldstein et al., 2013) and the presence of withdrawal symptoms have been noted in a subset of human cocaine users (Brower et al., 1988; Sofuoglu et al., 2003). Withdrawal was not assessed as part of the current experiments, but would be a useful feature for future studies, particularly as it may describe an additional source of individual variability. Finally, sleep/waking was assessed exclusively through EEG/EMG, which while the gold standard for sleep/waking state assessment, does not provide information about neuronal activity, the neurochemical environment, nor any indication of intracellular activity such as gene transcription or receptor trafficking, which, amongst other dynamic processes, would underlie any functional effect of altered sleep/waking behavior. Assessment of orexin neuronal activity in cocaine and saline treated animals would be of particular interest given the hypothesized role for orexin as an intermediator of arousal and motivated behaviors (Tyree et al., 2018).

## Conclusion

Chronic, repeated cocaine increased arousal throughout the course of the 13 days injection period with increased sleep latency and gamma energy persisting up to 2 weeks into forced abstinence. The homeostatic response to sleep loss was maintained as determined by the unchanged SWS SWE following externally enforced sleep deprivation and pharmacologically enforced arousal. These results indicate that sleep disturbance is likely due to increases in arousal that is especially prominent at the end of the active phase in mice as opposed to decreases in sleep pressure. Furthermore, this suggests that a most promising therapeutic target for severe stimulant abuse is arousal reduction. Orexin receptor antagonism improves sleep-related outcomes in a population of individuals with cocaine-use disorder (Suchting et al., 2020) providing preliminary support for this approach.

## Data Availability Statement

The raw data supporting the conclusions of this article will be made available by the authors, without undue reservation.

## Ethics Statement

The animal study was reviewed and approved by Institutional Animal Care and Use Committee, North Texas VA Health Care System.

## Author Contributions

TB performed the experiments and analyzed data. Both authors designed the experiments, wrote the manuscript, and approved the submitted version.

## Author Disclaimer

The contents do not represent the views of the U.S. Department of Veterans Affairs or the United States Government.

## Conflict of Interest

The authors declare that the research was conducted in the absence of any commercial or financial relationships that could be construed as a potential conflict of interest.

## Publisher’s Note

All claims expressed in this article are solely those of the authors and do not necessarily represent those of their affiliated organizations, or those of the publisher, the editors and the reviewers. Any product that may be evaluated in this article, or claim that may be made by its manufacturer, is not guaranteed or endorsed by the publisher.

## Supplementary Material

Supplementary Figure 1 **(A)** Cocaine increased SL following each daily injection. **(B)** Cocaine increased REML following each daily injection. Cocaine *n* = 10 (one mouse average did not include day 7), saline *n* = 9 (two mice averages did not include day 7). The dashed line indicates the 1 h between successive injections. Asterisks above lines indicate significant difference between groups at those timepoints. *P* and *F* values and statistics for all pairwise comparisons with significant differences or trends toward significance are given in Supplementary Table 1.

Supplementary Figure 2 Heat map of % time in waking (top), % SWS (middle), % REM (bottom) across each of the 4 baseline days and 13 injection days. The relative increase in waking following cocaine **(A)** and relative decrease in SWS and REM compared to the same time during baseline is readily noticeable as is the delayed recovery in the cocaine group, while the time in state between baseline and saline **(B)** shows comparatively similar colors. Note that the heat map scale range varies across states, but is consistent across groups. Cocaine *n* = 10 (one mouse missing day 7), saline *n* = 9 (two mice missing day 7). Yellow lines to the left of each graph indicate the light phase.

Supplementary Figure 3 **(A)** Alterations in the circadian distribution of waking compared to baseline continued into the post-injection/forced abstinence period following cocaine exposure with additional point differences in SWS and REM within the post-injection period (*n* = 5–6 [one mouse excluded p22–35]). **(D)** Saline exposure induced a change in % waking across bins from baseline that persisted into the post-injection period, along with a non-significant change in % SWS across bins from baseline, and altered the distribution of REM during the post-injection period (*n* = 6). There was no cumulative difference in any state between cocaine and saline groups during the early (**B**, p1–14) and late (**C**, p22–35) post-injection/forced abstinence period (cocaine *n* = 5, saline *n* = 6). Asterisks above symbols indicate a within-group difference from baseline at that timepoint. *P* and *F* values and statistics for all pairwise comparisons with significant difference or trends toward significance are given in Supplementary Table 2. The yellow bar indicates the light phase.

Supplementary Figure 4 **(A)** Though not different from saline levels, an apparent dip in sleep intensity within the circadian range in which cocaine was previously administered persists into the early portion of the post-injection/forced abstinence period. **(B)** The dip in SWS SWA in cocaine experienced animals remains visible, but muted during the later portion of the post-injection/forced abstinence period. **(C)** There was no difference in average SWS SWA between groups or compared to baseline levels during both the early and late portion of the post-injection/forced abstinence period (cocaine *n* = 5–6 [one mouse excluded in p22–35], saline *n* = 6). **(D)** SWS SWE, SWS time, SWS SWS % baseline did not differ between groups, while SWS SWE and SWS % time was increased in saline-treated animals above baseline at discrete points during the post-injection/forced abstinence period (cocaine *n* = 5–6 [one mouse exclude p22–35], saline *n* = 6). **(E)** As with SWS metrics, W metrics did not vary by group during the post-injection/forced abstinence period, although group by time interactions were present W SWE and W SWA with groups appearing to diverge during the latter portion of the post-injection/forced abstinence period. Further, average W SWE and W SWA was increased above baseline at discrete points in both groups while average W time was decreased below baseline in saline-experienced animals during the latter portion of the post-injection/forced abstinence period cocaine *n* = 5–6 [one mouse exclude p22–35], saline *n* = 6). Dashed lines at 100 indicates baseline level, asterisks within bars indicate a within-group difference from baseline at those timepoints and conditions. The dashed line at 100 indicates baseline level. *P* and *F* values and statistics for all pairwise comparisons with significant difference or trends toward significance are given in Supplementary Table 5.

Supplementary Figure 5 **(A)** Both cocaine- (left) and saline- (right) experienced animals showed increased delta/theta ratio during discrete points across the 24 h period; however, these increases were more extensive across the 24 h period and from the early to late post-injection/forced abstinence period in saline-treated animals (cocaine *n* = 5–6 [one mouse excluded p22–35], saline *n* = 6). **(B)** Neither cocaine nor saline-experienced animals showed a significant difference in W episode duration as a % of baseline, though saline-experienced animals showed a non-significant trend toward a difference (cocaine *n* = 5–6 [one mouse excluded p22–35], saline *n* = 6). Cocaine-experienced animals showed an increase in average W episode duration compared to baseline during the end of the light phase late in the post-injection/forced abstinence period, while saline-experienced animals showed a decrease in average W episode duration compared to baseline during the beginning of the dark phase across the post-injection/forced abstinence period. Dashed lines at 100 indicates baseline level, asterisks above lines indicate a within-group difference between those timepoints, asterisks and pound symbols within bars indicate a significant and non-significant difference from baseline at those timepoints, respectively, yellow lines indicate lights on. *P* and *F* values and statistics for all pairwise comparisons with significant difference or trends toward significance are given in Supplementary Table 6.

Supplementary Figure 6 Cocaine experience did not influence arousal following sleep deprivation as determined by SL measures **(A)**, SL to a stable sleep period **(B)**, REML **(C)**, and gamma energy during waking relative to baseline measures [**(D)**; cocaine *n* = 6, saline *n* = 5]. *P* and *F* values and statistics for all pairwise comparisons with significant difference or trends toward significance are given in Supplementary Table 7.
