# Supplementary figures and images for: Arousal-Mediated Sleep Disturbance Persists During Cocaine Abstinence in Male Mice
Source: Front Neurosci. 2022 Jun 23;16:868049. doi: 10.3389/fnins.2022.868049 (PMC9260276; doi:10.3389/fnins.2022.868049)

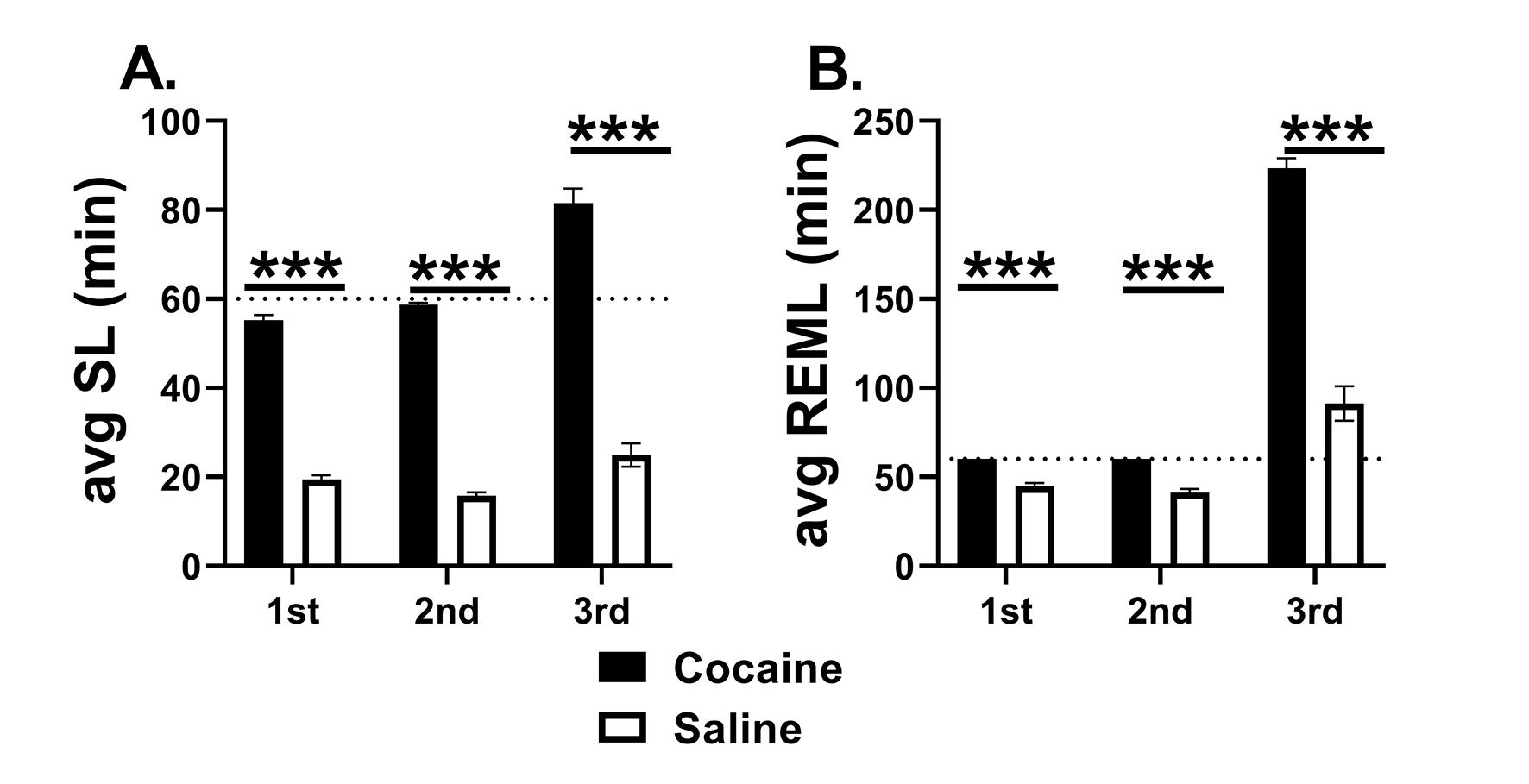

Supplement: Supplementary Figure 1 — (A) Cocaine increased SL following each daily injection. (B) Cocaine increased REML following each daily injection. Cocaine n = 10 (one mouse average did not include day 7), saline n = 9 (two mice averages did not include day 7). The dashed line indicates the 1 h between successive injections. Asterisks above lines indicate significant difference between groups at those timepoints. P and F values and statistics for all pairwise comparisons with significant differences or trends toward significance are given in Supplementary Table 1. [file Image_1.TIF]

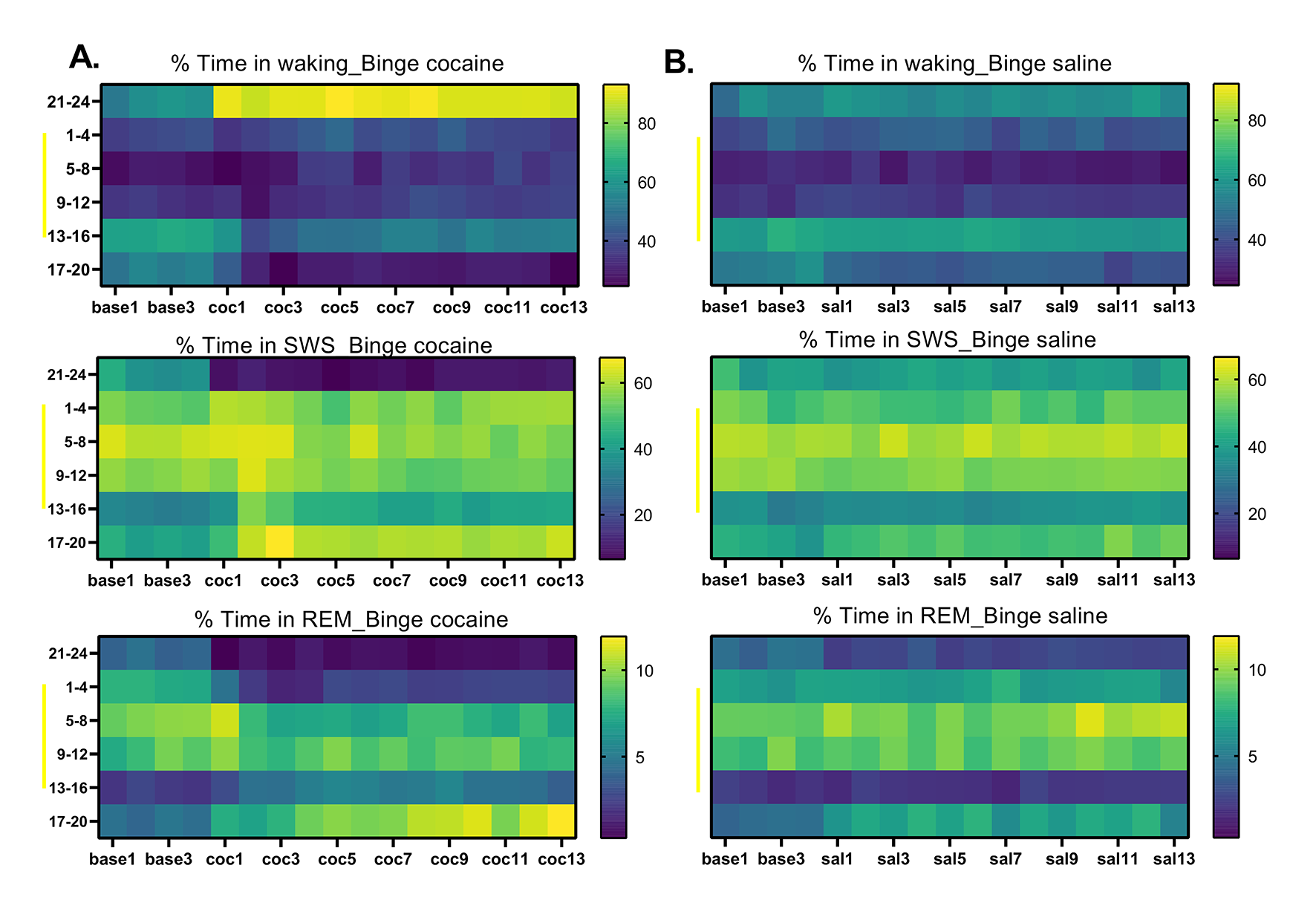

Supplement: Supplementary Figure 2 — Heat map of % time in waking (top), % SWS (middle), % REM (bottom) across each of the 4 baseline days and 13 injection days. The relative increase in waking following cocaine (A) and relative decrease in SWS and REM compared to the same time during baseline is readily noticeable as is the delayed recovery in the cocaine group, while the time in state between baseline and saline (B) shows comparatively similar colors. Note that the heat map scale range varies across states, but is consistent across groups. Cocaine n = 10 (one mouse missing day 7), saline n = 9 (two mice missing day 7). Yellow lines to the left of each graph indicate the light phase. [file Image_2.tif]

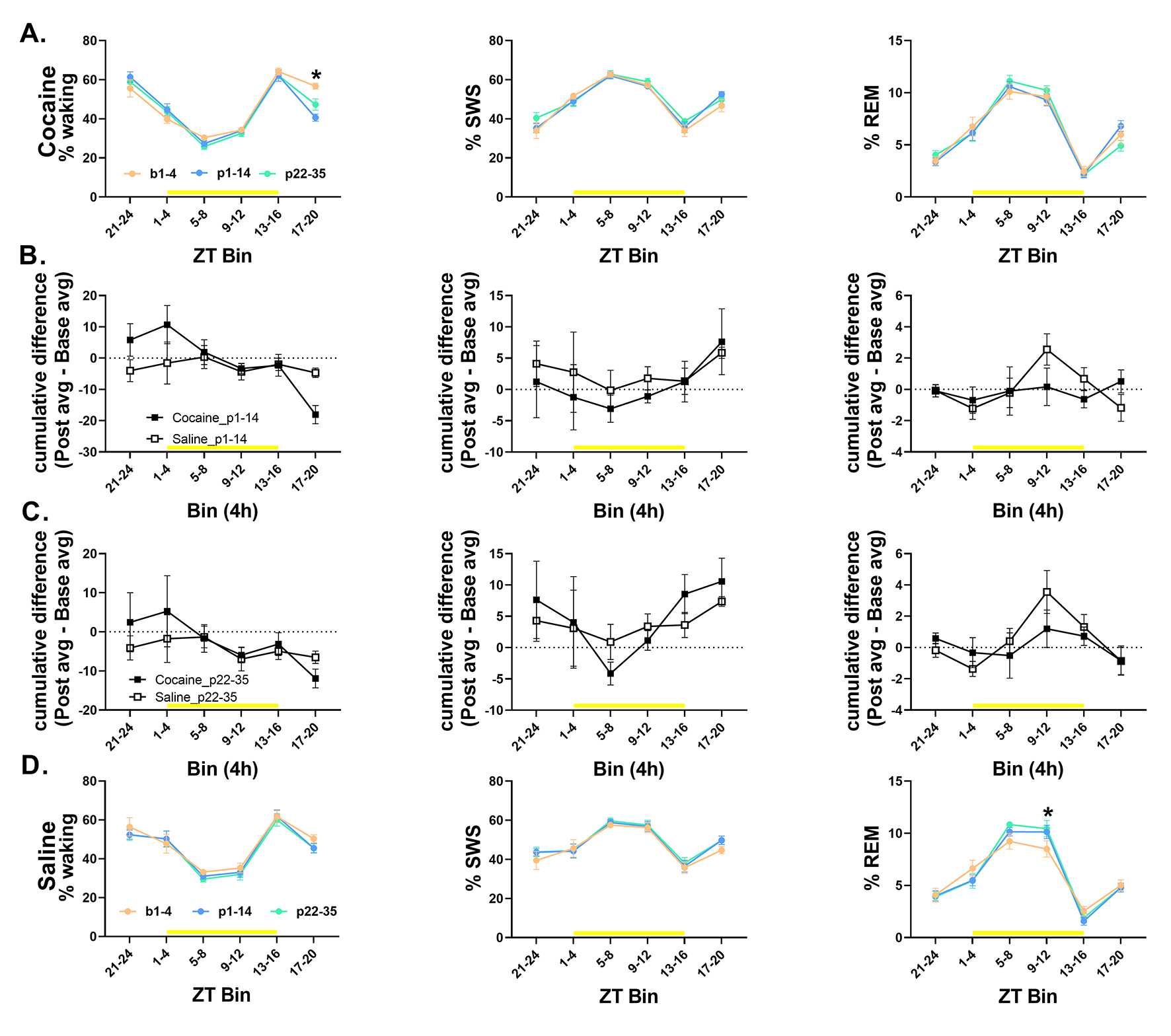

Supplement: Supplementary Figure 3 — (A) Alterations in the circadian distribution of waking compared to baseline continued into the post-injection/forced abstinence period following cocaine exposure with additional point differences in SWS and REM within the post-injection period (n = 5–6 [one mouse excluded p22–35]). (D) Saline exposure induced a change in % waking across bins from baseline that persisted into the post-injection period, along with a non-significant change in % SWS across bins from baseline, and altered the distribution of REM during the post-injection period (n = 6). There was no cumulative difference in any state between cocaine and saline groups during the early (B, p1–14) and late (C, p22–35) post-injection/forced abstinence period (cocaine n = 5, saline n = 6). Asterisks above symbols indicate a within-group difference from baseline at that timepoint. P and F values and statistics for all pairwise comparisons with significant difference or trends toward significance are given in Supplementary Table 2. The yellow bar indicates the light phase. [file Image_3.TIF]

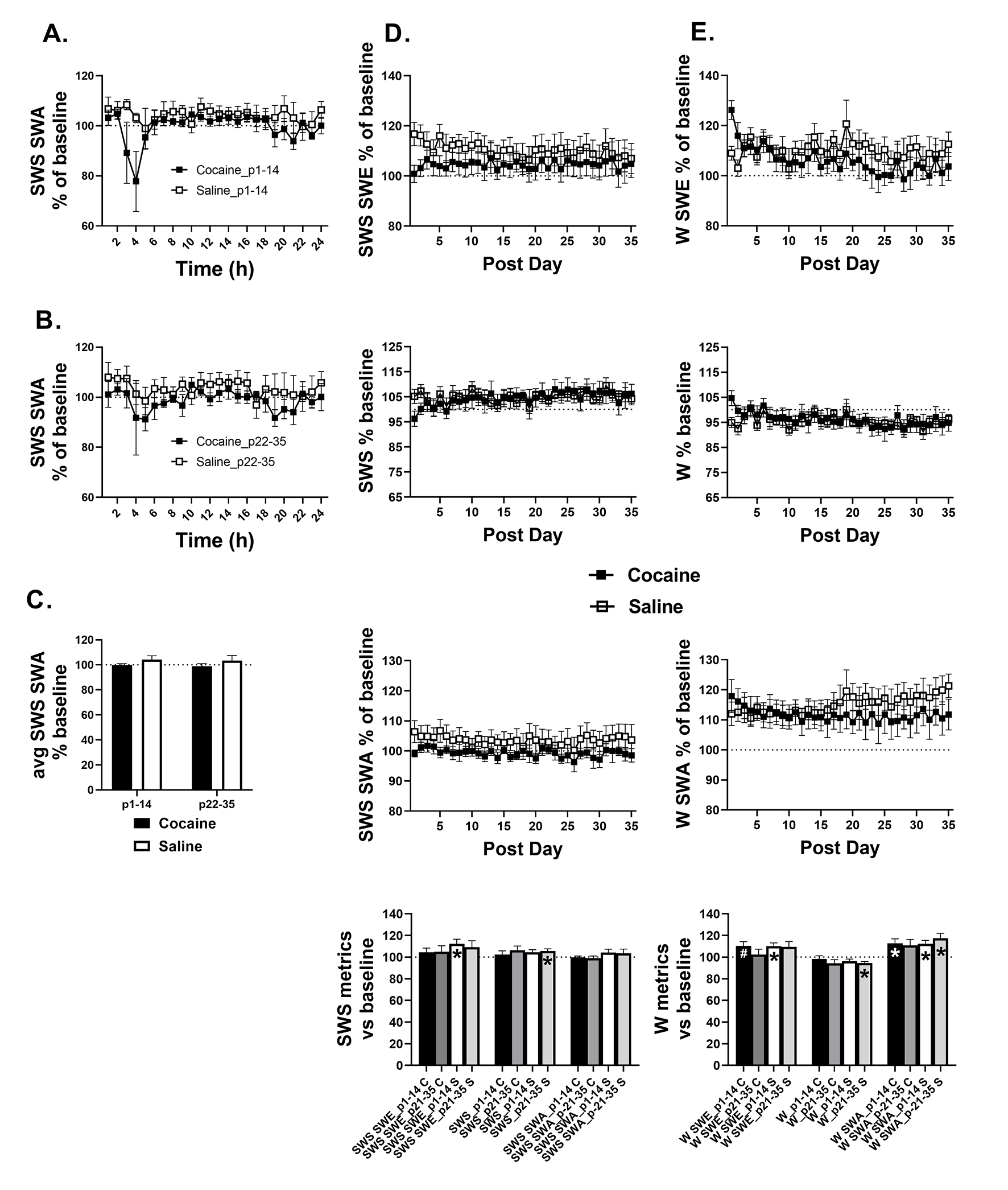

Supplement: Supplementary Figure 4 — (A) Though not different from saline levels, an apparent dip in sleep intensity within the circadian range in which cocaine was previously administered persists into the early portion of the post-injection/forced abstinence period. (B) The dip in SWS SWA in cocaine experienced animals remains visible, but muted during the later portion of the post-injection/forced abstinence period. (C) There was no difference in average SWS SWA between groups or compared to baseline levels during both the early and late portion of the post-injection/forced abstinence period (cocaine n = 5–6 [one mouse excluded in p22–35], saline n = 6). (D) SWS SWE, SWS time, SWS SWS % baseline did not differ between groups, while SWS SWE and SWS % time was increased in saline-treated animals above baseline at discrete points during the post-injection/forced abstinence period (cocaine n = 5–6 [one mouse exclude p22–35], saline n = 6). (E) As with SWS metrics, W metrics did not vary by group during the post-injection/forced abstinence period, although group by time interactions were present W SWE and W SWA with groups appearing to diverge during the latter portion of the post-injection/forced abstinence period. Further, average W SWE and W SWA was increased above baseline at discrete points in both groups while average W time was decreased below baseline in saline-experienced animals during the latter portion of the post-injection/forced abstinence period cocaine n = 5–6 [one mouse exclude p22–35], saline n = 6). Dashed lines at 100 indicates baseline level, asterisks within bars indicate a within-group difference from baseline at those timepoints and conditions. The dashed line at 100 indicates baseline level. P and F values and statistics for all pairwise comparisons with significant difference or trends toward significance are given in Supplementary Table 5. [file Image_4.TIF]

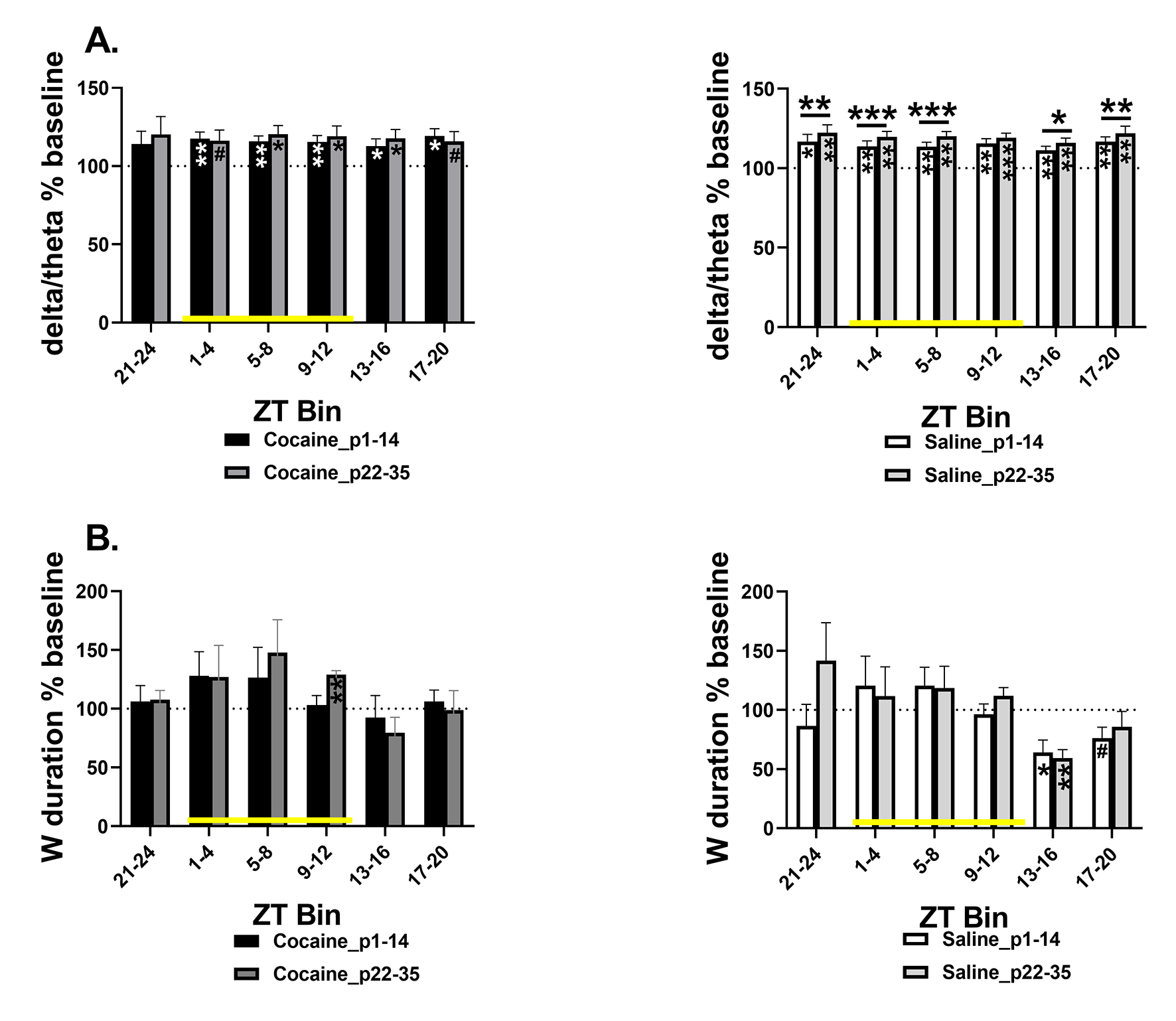

Supplement: Supplementary Figure 5 — (A) Both cocaine- (left) and saline- (right) experienced animals showed increased delta/theta ratio during discrete points across the 24 h period; however, these increases were more extensive across the 24 h period and from the early to late post-injection/forced abstinence period in saline-treated animals (cocaine n = 5–6 [one mouse excluded p22–35], saline n = 6). (B) Neither cocaine nor saline-experienced animals showed a significant difference in W episode duration as a % of baseline, though saline-experienced animals showed a non-significant trend toward a difference (cocaine n = 5–6 [one mouse excluded p22–35], saline n = 6). Cocaine-experienced animals showed an increase in average W episode duration compared to baseline during the end of the light phase late in the post-injection/forced abstinence period, while saline-experienced animals showed a decrease in average W episode duration compared to baseline during the beginning of the dark phase across the post-injection/forced abstinence period. Dashed lines at 100 indicates baseline level, asterisks above lines indicate a within-group difference between those timepoints, asterisks and pound symbols within bars indicate a significant and non-significant difference from baseline at those timepoints, respectively, yellow lines indicate lights on. P and F values and statistics for all pairwise comparisons with significant difference or trends toward significance are given in Supplementary Table 6. [file Image_5.TIF]

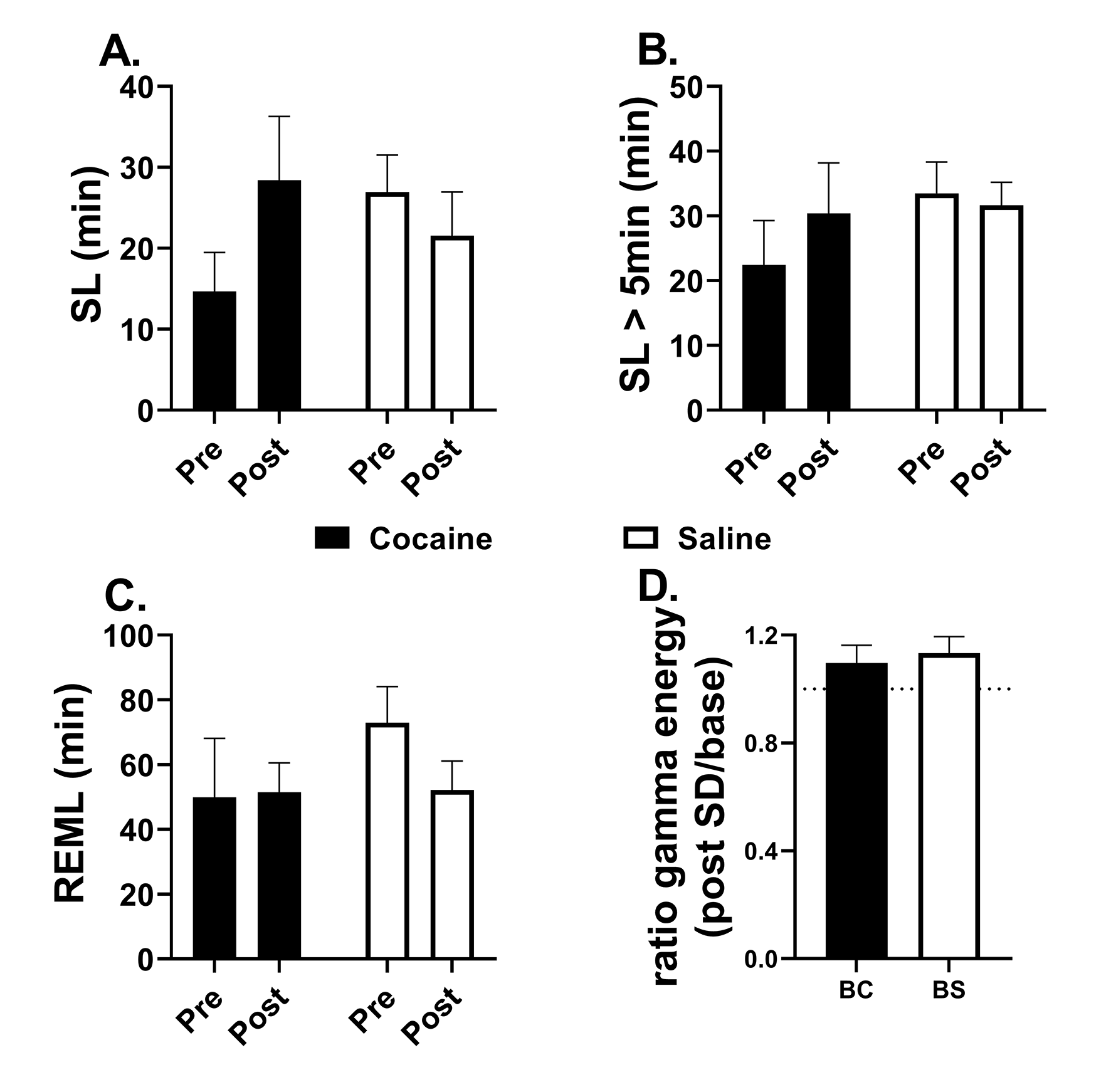

Supplement: Supplementary Figure 6 — Cocaine experience did not influence arousal following sleep deprivation as determined by SL measures (A), SL to a stable sleep period (B), REML (C), and gamma energy during waking relative to baseline measures [(D); cocaine n = 6, saline n = 5]. P and F values and statistics for all pairwise comparisons with significant difference or trends toward significance are given in Supplementary Table 7. [file Image_6.TIF]
